# 

**Published:** 1905-10-15

**Authors:** 


### CONTENTS.

Event and Comment - - - - - - - - - 487

The Need of Oral Prophylaxis and Some Results Obtained by It,
By Edward B. Spalding, D.D.S. 492

Pyorrhea Alveolaris, - - - By C. A. Rutbridge, D.D.S. 503

Fatalities - - - - - - - - - - - - 532

Indents - - - - - - - - - - - - 533

Announcements - - - - - - - - - - 538

